# Differentiation of Dental Pulp Stem Cells into Neuron-Like Cells in Serum-Free Medium

**DOI:** 10.1155/2013/250740

**Published:** 2013-11-18

**Authors:** Shahrul Hisham Zainal Ariffin, Shabnam Kermani, Intan Zarina Zainol Abidin, Rohaya Megat Abdul Wahab, Zulham Yamamoto, Sahidan Senafi, Zaidah Zainal Ariffin, Mohamad Abdul Razak

**Affiliations:** ^1^School of Biosciences and Biotechnology, Faculty of Science and Technology, Universiti Kebangsaan Malaysia, 43600 Bangi, Selangor, Malaysia; ^2^Department of Orthodontics, Faculty of Dentistry, Universiti Kebangsaan Malaysia, Jalan Raja Muda Abdul Aziz, 50300 Kuala Lumpur, Malaysia; ^3^School of Biology, Faculty of Applied Science, Universiti Teknologi MARA, 40450 Shah Alam, Selangor, Malaysia; ^4^Allianze University College of Medical Sciences, Waziria Medical Square, Jalan Bertam 2, 13200 Kepala Batas, Penang, Malaysia

## Abstract

Dental pulp tissue contains dental pulp stem cells (DPSCs). Dental pulp cells (also known as dental pulp-derived mesenchymal stem cells) are capable of differentiating into multilineage cells including neuron-like cells. The aim of this study was to examine the capability of DPSCs to differentiate into neuron-like cells without using any reagents or growth factors. DPSCs were isolated from teeth extracted from 6- to 8-week-old mice and maintained in complete medium. The cells from the fourth passage were induced to differentiate by culturing in medium without serum or growth factors. RT-PCR molecular analysis showed characteristics of *Cd146^+^*, *Cd166^+^*, and *Cd31^−^* in DPSCs, indicating that these cells are mesenchymal stem cells rather than hematopoietic stem cells. After 5 days of neuronal differentiation, the cells showed neuron-like morphological changes and expressed MAP2 protein. The activation of *Nestin* was observed at low level prior to differentiation and increased after 5 days of culture in differentiation medium, whereas *Tub3* was activated only after 5 days of neuronal differentiation. The proliferation of the differentiated cells decreased in comparison to that of the control cells. Dental pulp stem cells are induced to differentiate into neuron-like cells when cultured in serum- and growth factor-free medium.

## 1. Introduction

Dental pulp tissue contains many types of cells including committed cells (e.g., endothelial cells) and uncommitted cells (i.e., DPSCs). DPSCs are of mesenchymal stem cells (MSCs) [1]. In mice, the majority of MSCs were isolated from bone marrow [2] and peripheral blood [3, 4]. These MSCs can be characterized by the expression of specific gene markers such as *CD44*, *CD73*, *CD90*, *CD105*, *CD117*, and *CD166* [5, 6].

DPSCs are capable of differentiating into multilineage cells [7–9] including neuron-like cells [10]. Neuron-like cells differentiated from MSCs derived from bone marrow cells [11–13] and brain [14]. However, MSCs derived from dental pulp, that is, DPSCs, are also capable of differentiating into neuron-like cells [10]. The characteristics of MSCs from bone marrow are similar to those cells derived from dental pulp [11]. Both types of MSCs express *Cd44*, *Cd106*, *Cd146*, and *Cd166* [15–17].

Many factors are involved in neuronal differentiation including nestin [18], tubulin3 (Tub3) [19], and MAP2 [20]. Nestin is involved in the radial growth of axons during neuronal differentiation in vertebrate cells [19, 21]. Therefore, Nestin is known as a neural marker and its presence can be considered as a criterion for the ability to differentiate into neurons [18, 22]. However, Nestin has shown to be expressed by other cell types such as hair follicle stem cells [23], pericytes [24], endothelial cells [25], myofibroblasts, and pancreatic fibroblasts [26]. Therefore, analysis on expression of other specific neuron markers such as Tub3 [27, 28] and MAP2 [29, 30] has been done concurrently for neuronal confirmation. Tub3 and MAP2 play a role in the stability of axons and neuronal cell bodies [20, 31]. Certain growth factors, such as epidermal growth factor, basic fibroblast growth factor, and retinoic acid, were used for neuronal induction [32–35]. Dimethyl sulfoxide (DMSO) was also used to induce transformation of MSCs into neuron-like phenotypes *in vitro *[12, 13]. The objective of the present study was to examine the directed differentiation of DPSCs into neuron-like cells in the absence of chemical induction. 

## 2. Materials and Methods

### 2.1. Isolation of Dental Pulp Cells

Incisor teeth were extracted from 6- to 8-week-old mice under sterile conditions and placed in medium containing 1X PBS (Sigma, USA). The extracted dental pulp was washed with 1X PBS containing 1% (v/v) penicillin-streptomycin (Invitrogen, USA).

Dental pulp tissue was incubated for 1 hour in 4-unit collagenase type I at 37°C, followed by several rounds of enzymatic disaggregation. The cells were centrifuged at 1200 g for 10 minutes at 25°C and cultured in complete medium consisting of *α*-MEM (Invitrogen, USA) supplemented with 20% (v/v) FBS (Biowest, USA) and 1% (v/v) penicillin-streptomycin. The cells 1 × 10^5^ cells/mL obtained were put in a T25 flask containing complete medium and cultured in an incubator with 5% CO_2_ atmosphere and 95% humidity at 37°C.

After 24 hours, the suspended cells were removed from the medium, and the flask was washed with 1X PBS solution. The cells were grown in complete medium until 80% confluency. A solution of 0.25% (v/v) trypsin-EDTA (Sigma, USA) was used to detach the dental pulp cells from the flask surface for subculturing in another flask at 1 × 10^5^ cells/mL. For cryopreservation and storage, cells at the fourth passage were placed in cryovials containing freezing medium consisting of *α*-MEM, 10% (v/v) DMSO (Sigma, USA), and 50% (v/v) FBS and stored in liquid nitrogen. In this study, the cells used were at the fourth passage.

### 2.2. Differentiation of Dental Pulp Stem Cells into Neuronal Cells

Approximately 1 × 10^5^ cells were transferred into 24-well plates containing complete medium and were allowed to grow for 24 hours until adherence. The medium was discarded, and the cells were washed with PBS. The cells were then cultured in serum- and growth factor free-medium consisting of *α*-MEM and 1% (v/v) penicillin-streptomycin for 5 days. The medium was changed every 2-3 days during this 5-day period. As a control, the same number of cells was cultured in complete medium (consists of *α*-MEM, 1% (v/v) penicillin-streptomycin, and 15% (v/v) fetal bovine serum) for 5 days. Cell morphology was monitored on days 2 and 5 of neuronal differentiation using an Olympus phase-contrast microscope.

### 2.3. Molecular Analysis Using RT-PCR

Control and differentiated cells were detached using 0.25% (v/v) trypsin-EDTA and centrifuged at 1400 g for 10 minutes. Total RNA was extracted using TRI-reagent (Sigma, USA) according to the manufacturer's protocol. The purity of the total RNA was assessed spectrophotometrically at 260 and 280 nm, with an A_260_ : A_280_ ratio of 1.8–2.0 considered acceptable. Approximately, 300 ng of total RNA sample was used for each RT-PCR reaction with the Access Quick RT-PCR system kit (Promega, USA). The primers were designed using the Primer Premier 5.0 software program based on sequences obtained from NCBI. Information on the primers is summarized in Table 1.

Primary cDNA synthesis was performed using AMV reverse transcriptase for 45 minutes at 45°C followed by deactivation for 2 minutes at 94°C. The amplification consisted of denaturation for 30 seconds at 94°C, annealing for 60 seconds, and extension for 60 seconds at 68°C, performed for 40 cycles. A final extension step was performed for 7 minutes at 68°C. The amplified products were separated using 1% (w/v) agarose gel electrophoresis, stained, and analysed. The amplified products were subjected to DNA sequencing and verified using the BLASTN program from NCBI. As for expression level analysis, a total of 1 *μ*g of total RNA was used for each amplification and intensity was determined using online ImageJ 1.47 program (http://rsbweb.nih.gov/ij/). 

### 2.4. Immunocytochemistry of MAP2

The induced neuronal cells were washed with 1X PBS and fixed at 4°C with 4% (v/v) paraformaldehyde for 2 hours. The cells were washed 3 times with 0.05% PBS-Tween 20 followed by 2% PBS-Triton-X for 10 minutes. Then, the cells were washed again 3 times with 0.05% PBS-Tween 20. The cells were incubated at 37°C with 10% goat serum diluted in 0.05% (v/v) PBS-Tween 20 plus 0.01 mg/mL BSA for 30 minutes prior to incubation of primary antibody. This solution was removed and primary antibody at 1 : 200 diluted with 0.05% (v/v) PBS-Tween 20 plus 3% (v/v) goat serum and 1 mg/mL BSA were added for 24 hours at 4°C. Then, the cells were washed 3 times with 0.05% (v/v) PBS-Tween 20 followed by incubation of secondary antibody anti-mouse IgG-FITC for 30 minutes at room temperature diluted at 1 : 50 with Tween 20 plus 3% (v/v) goat serum and 1 mg/mL BSA. Finally, the cells were washed 3 times with 0.05% (v/v) PBS-Tween 20 prior to analysis using fluorescence microscope. Negative control used was undifferentiated cells without neuronal induction, that is, cultured in complete medium. 

### 2.5. 3-(4,5-Dimethylthiazol-2-yl)-2,5-diphenyltetrazolium Bromide (MTT) Assay

Approximately 1 × 10^4^ cells were seeded in 96-well plates and incubated for 24 hours at 37°C. Two groups of cells were cultured: one in complete medium (control) and the other in serum- and growth factor-free medium. After, 24-hour incubation, approximately 20 *μ*L MTT (5 mg/mL) was added to each sample and the samples were incubated for another 4h at 37°C. The mixture was slowly removed, 200 *μ*L of DMSO was added to each well, and the wells were measured by an ELISA Plate Reader at 570 nm. Each experiment was repeated in triplicate. The cells were subjected to MTT assay on the first, third, and fifth days of neuronal induction. The number of viable cells for each analysis was determined using a standard graph created prior to the experiment.

### 2.6. Statistical Analysis

Data from the differentiated and control groups were compared using paired *t*-test in SPSS program version 16.0.2. Differences with a *P* value < 0.05 were considered statistically significant. Data obtained were presented as average (mean ± SD; standard deviation) from three independent experiments (*n* = 3).

## 3. Results

### 3.1. Identification of Mesenchymal Stem Cells in Dental Pulp Tissue

**Figure 1:**
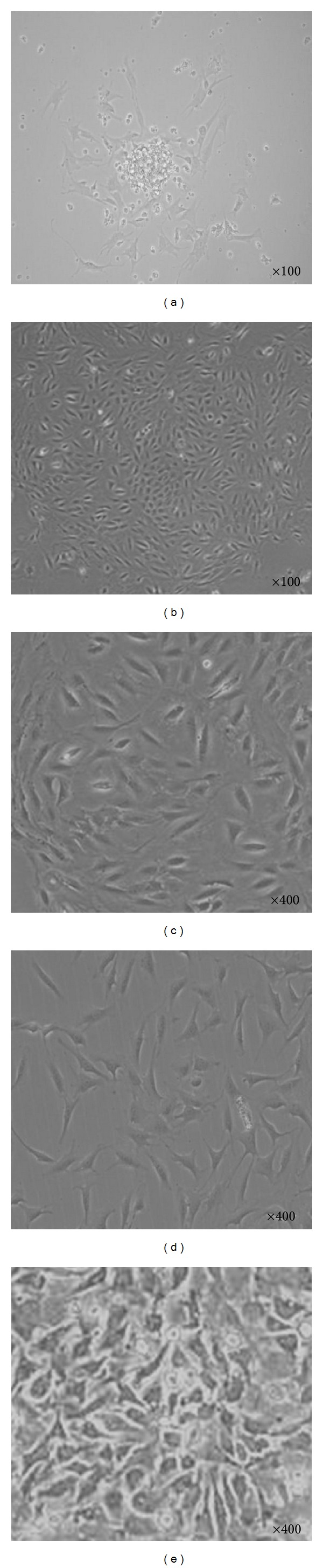
Characteristics of isolated and *in vitro* mouse dental pulp stem cells. Colonies derived from dental pulp at the first passage (a) and after 24 hours of culture (b). Colonies began to show changes in shape after the second passage (c), a fibroblastic cell shape after the fourth passage (d), and confluence after 2-3 days of culture in complete medium (e).

The identity of dissociated cells isolated from dental pulp tissue using collagenase was confirmed by their capacity to form adherent colonies consisting of sphere-like clusters of cells (Figure 1). Averages of 6.8 × 10^4^ cells/cm^2^ were found capable to obtain colonies after 24 hours cultured in the complete medium. Then, the suspended cells were discarded and only adherent cells were expanded in the medium. The suspended cells may have been cells that were unable to survive in the medium. The colonies began to change their shape during the second passage. The cells assumed a fibroblast-like morphology with a long, thin body during the fourth passage and became confluent after 2 to 3 days of *in vitro* culture in complete medium.

**Figure 2:**
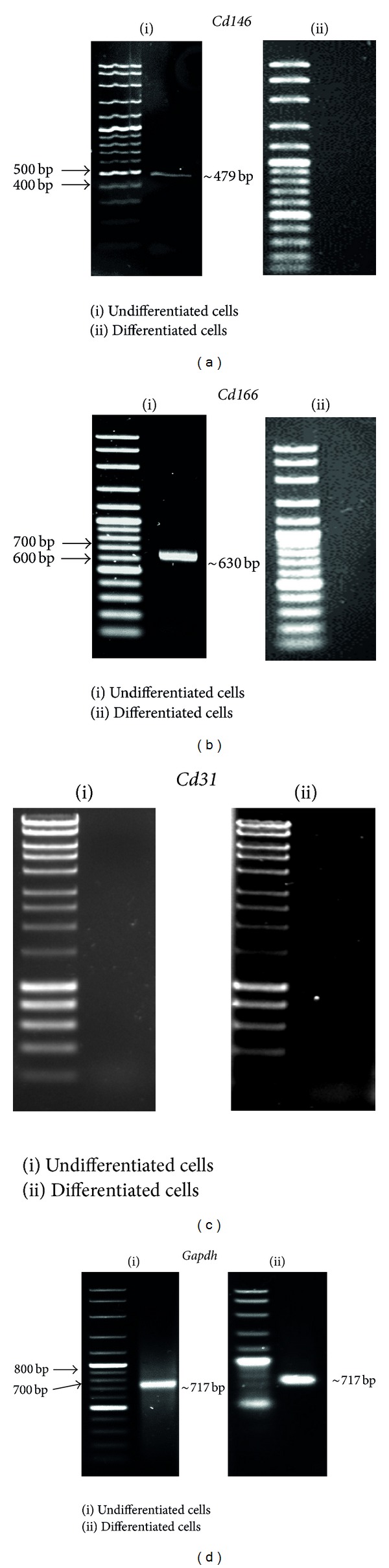
Activation of mesenchymal stem cell markers. The activation of *Cd146* (~479 bp) (a) and *Cd166 *(~630 bp) (b) was observed only in cells before differentiation, indicating that the cells were mesenchymal stem cells. *Cd31* was inactivated before and after neuronal differentiation (c). *Gapdh* (~717 bp), a housekeeping gene, was expressed before and after differentiation (d). Panel (i) is representing undifferentiated cells, that is, cells before neuronal differentiation, while panel (ii) is representing differentiated cells, that is, cells after neuronal differentiation.

Molecular analysis was performed to validate the types of cells in the fourth passage. The total RNA was extracted from the fourth passage of dental pulp cells and was subjected to RT-PCR analysis (Figure 2). This analysis showed *Cd146* and *Cd166* amplicons in these cells, whereas activation of *Cd31* was not observed. The amplicon of *Gapdh* was found in dental pulp cells both before and after differentiation. Analysis of expression level for *Cd146* and *Cd166* was shown to produce 118.0 ± 16.8% and 77.5 ± 14.3%, respectively, when compared to *Gapdh* (100%) which was used to normalize the cellular mRNA level. 

### 3.2. Morphological Changes into Neuron-Like Cells and Expression of MAP2 Protein

**Figure 3:**
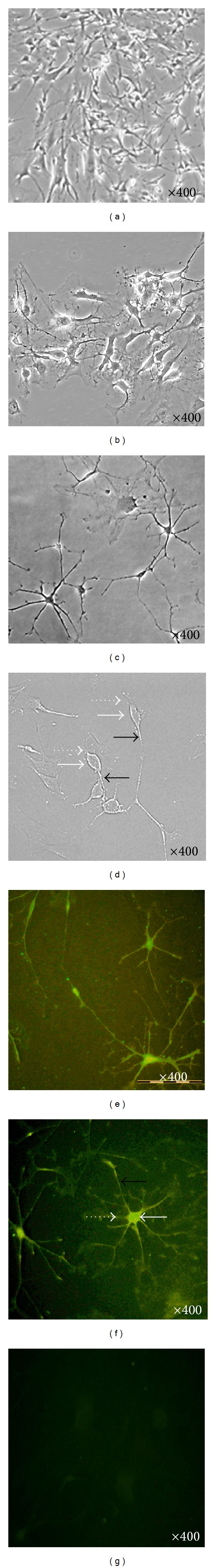
Characteristics of differentiated cells. Neuron-like cells appeared among the dental pulp stem cells ((a), (b)) 5 days after neuronal induction (c). The perikaryon, dendrites, and axons of neurons are indicated, respectively, by white arrows, open arrowheads, and black arrows (d). Immunofluorescence staining for neuron markers MAP2 was performed after 5 days of neuronal induction ((e), (f)). MAP2 marker was not detected in DPSCs without neuronal induction, that is, negative control (g).

After 5 days of culture, most of the cells showed a morphological change to a long, thin body shape (Figure 3). The cytoplasm was contracted toward the nucleus and assumed a multipolar shape. The cells displayed small, spherical, and contracted bodies and a conical cytoplasm with branches resembling the neuronal perikaryon, axon, and dendrite. The perikaryon, dendrite, and axon of a neuronal cell are indicated, respectively, by a white arrow, an open arrowhead, and a black arrow. Approximately 60%–70% of the cell population differentiate into neuron-like cells, an indication that differentiation occurred due to the absence of serum and growth factors but not because of spontaneous differentiation. DPSCs expressed the neuron-specific protein marker MAP2 after 5 days of culture in serum- and growth factor-free medium (Figure 3). However MAP2 was not detected in DPSCs or undifferentiated cells without neuronal induction, that is, cultured in complete medium (negative control) (Figure 3).

### 3.3. Activation of Neuronal Markers

**Figure 4:**
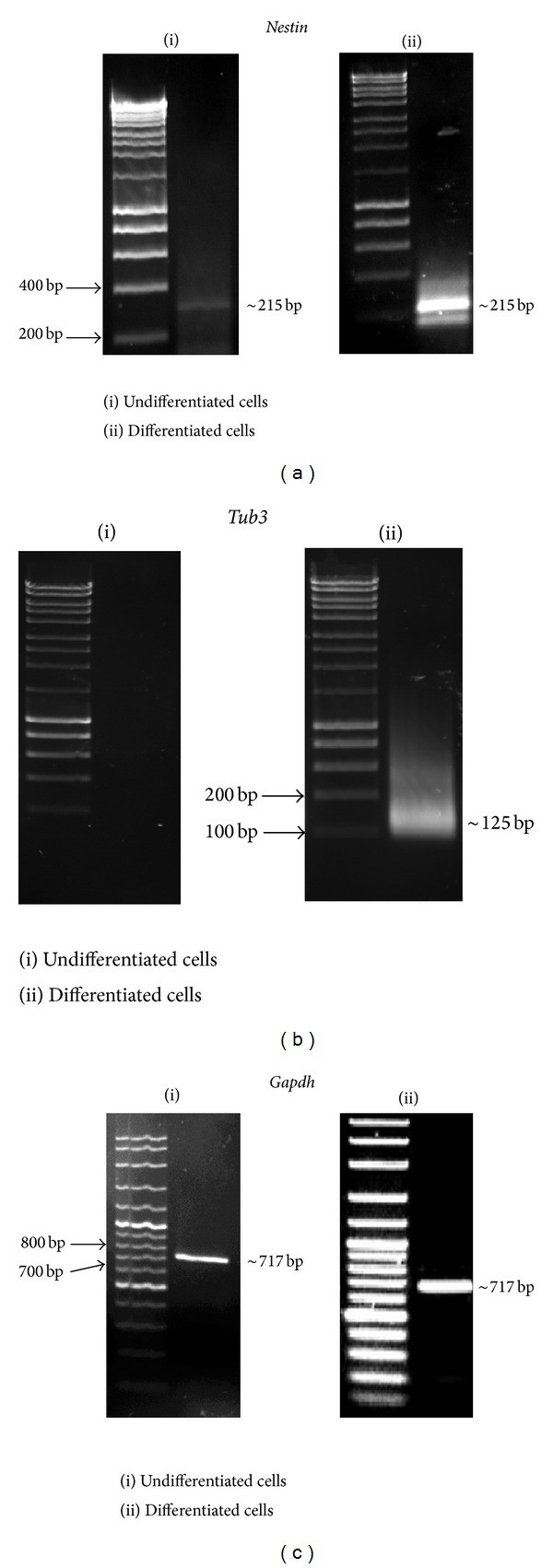
Activation of specific neuronal markers. The activation of *Nestin *(~215 bp) (a) and *Tub3 *(~125 bp) (b) indicated that the cells had differentiated into neurons. *Gapdh* (~717 bp), a housekeeping gene, was activated before and after differentiation (c). Panel (i) is representing undifferentiated cells, that is, cells before neuronal differentiation, while panel (ii) is representing differentiated cells, that is, cells after neuronal differentiation.

The cells showed expression of *Cd146* ^*+*^, *Cd166* ^*+*^, and *Cd31* ^*−*^ before differentiation. RT-PCR analysis showed the presence of a *Nestin* amplicon (~215 bp) in the cells, both before and after differentiation. However, the intensity of *Nestin* activation was significantly higher after differentiation compared with before differentiation (Figure 4(a)). An amplicon of *Tub3* (~125 bp) was observed after day 5 of neuronal differentiation (Figure 4(b)). *Tub3* was shown to be activated after 5 days of neuronal differentiation. *Gapdh* was used as a positive control both before and after differentiation. A *Gapdh* amplicon (~717 bp) was found in cells both before and after differentiation, indicating that *Gapdh* remains activated in both types of cells (Figure 4(c)). 

### 3.4. Proliferation of Dental Pulp Stem Cells during Differentiation

**Figure 5:**
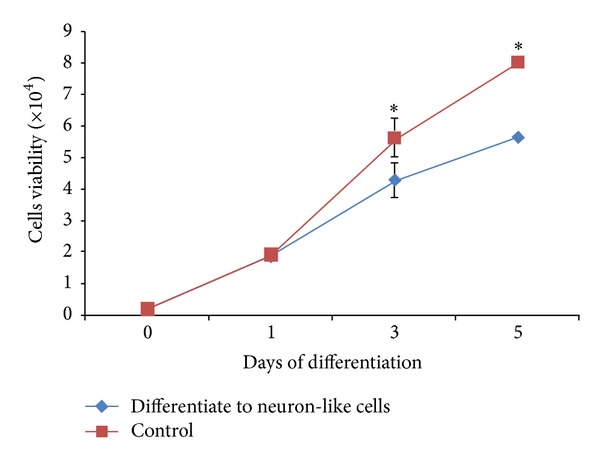
Viability of cells during directed differentiation. The viability of control (undifferentiated cells cultured in complete medium) versus differentiated cells cultured in serum- and growth factor-free medium. The numbers of both control and differentiated cells were significantly increased upon differentiation compared with day 0 of culture. The results are summarized as the mean ± SD. Statistical significance was determined using SPSS program version 16.0.2. *Statistical analysis showed significant differences (*P* < 0.05) of viable cells at days 3 and 5 of culture as compared to control.

Cell viability studies were performed to assess the proliferation capacity of both differentiated and undifferentiated cells during neuronal differentiation. The numbers of control and differentiated cells were significantly (*P* < 0.05) increased upon differentiation compared with day 0 of culture (Figure 5). However, the proliferation capacity of the cells began to change gradually after 24 hours of culture until day 5 of neuronal differentiation, with the number of control cells remaining higher. Both types of cells maintained their growth rate during the initial 24 hours, of culture. However, the differentiated cells showed a reduction of growth rate after 24 hours whereas the undifferentiated (control) cells maintained their growth rate, resulting in an increased number of viable cells. Statistical analysis showed a significant difference (*P* < 0.05) in cell number between the two types of cells at days 3 and 5 of culture (Figure 5). 

## 4. Discussion

The cells formed a fibroblast-like morphology during the fourth passage. Molecular analysis showing *Cd146* and *Cd166* amplicon but not *Cd31* validated that the fibroblast-like cells were MSCs rather than hematopoietic stem cells. *Cd146* and *Cd166* are mesenchymal stem cell markers [36, 37]. *Cd146 *is an early mesenchymal stem cell marker expressed within dental pulp tissues [36]. *Cd166* is a cell adhesion molecule that plays important roles in tight cell-to-cell interaction and in the regulation of MSCs differentiation [38]. While *Cd166* is expressed in a wide variety of tissues, it is usually restricted to subsets of cells involved in processes of dynamic growth and/or migration, including neural development and immune response [39]. The amplicon of *Gapdh* found in DPSC both before and after differentiation indicated that *Gapdh* is expressed in both types of cells. 

To confirm the differentiation of DPSCs to a neuronal phenotype and to demonstrate that this differentiation was not an artefact, three analyses were performed: morphological changes, expression of MAP2, and activation of neuronal markers. After 2 days of culture, most of the cells resembled multipolar neuron. However, some fibroblast-like cells with spread-out morphology were still observed in the population. We suggest that DPSCs changed gradually and differentiated into neuron-like cells after 5 days when cultured in serum- and growth factor-free medium. DPSCs from various tissues differentiated into neuronal cells by displaying neuron morphology [40, 41], similar to our observations. Thus, morphological analysis indicated that DPSCs isolated from dental pulp tissue differentiated into neurons.

RT-PCR analysis showed the presence of a *Nestin* amplicon. *Nestin* is a neuron marker in adult rat and human brains [42]. Both *Nestin* and *Tub3* were also used as markers to investigate neuronal differentiation in the hippocampus of mice after DPSC implantation [43]. The expression of both *Nestin* and *Tub3* after differentiation indicated that the DPSCs differentiated into neuronal cells. Nestin is one of the intermediate filaments found in the cytoskeleton of vertebrate cells [19, 44]. *Nestin* expression was used to track the proliferation, migration, and differentiation of neuronal stem cells. *Tub3* is expressed in all eukaryotic cells. It contributes to microtubule stability in neuronal cell and plays a role in axonal transport. In the present study, *Tub3* was activated after 5 days of neuronal differentiation. *Gapdh* was used as a positive control both before and after differentiation. *Gapdh* is a housekeeping gene which has always been activated by all mammalian cells whether by undifferentiated or differentiated cells [45]. It was used to determine the RNA quality of isolated DPSCs and to normalize the levels of mRNAs. A *Gapdh* amplicon was found in cells both before and after differentiation, indicating that *Gapdh* remains activated in both types of cells. 

*Nestin* activation was increased during neuronal differentiation. *Nestin* is known to be expressed within fibrous dental pulp tissue, however, its expression continued to be detected by the majority of DPSCs following neuronal induction; that is, expression of *Nestin* was increased when cells differentiated into neuron [28]. MAP2 and *Tub3* are expressed only after neuronal differentiation and are therefore utilized as markers of mature neuronal cells during the final stages of growth [46–48]. MAP2-positive neuronal cells have been shown to be adult neuronal cells produced by neuronal induction. Varied expression of *Nestin* in several cell types such as hair follicle stem cells, pericytes, endothelial cells, myofibroblasts, and pancreatic fibroblasts makes *Nestin* not specific to neuron [23–26]. However, combination of *Nestin* expression together with the expression of other neural markers, that is, *Tub3* and MAP2, can support neuronal differentiation. In addition, characterization of the neuron-like cell by morphological analysis also showed a positive result. 

While growth factors such as FGF and retinoic acid have been employed in previous studies, neuronal induction was induced in the present study solely by excluding serum and growth factors from the complete medium. Our observations on *Tub3 *and *Nestin* activation, neuron-like cells morphology, and MAP2 expression provide evidence for the directed differentiation of mouse dental pulp stem cells into neuron-like cells in serum- and growth factor-free medium. 

The proliferation of the differentiated cells continued although their growth rate was lower than that of the control group. Theoretically, proliferation and differentiation of cells cannot occur simultaneously. The signalling cues that coordinate these two processes are largely unknown. However, cell differentiation and proliferation are regulated simultaneously but independently, that cells often start differentiating long before they stop dividing, and that the initiation of differentiation is not restricted to any particular segment of the cell cycle [49, 50]. In the present study, differentiated cells began to divide slowly after 24 hours of culture and continued until the end of the differentiation period, suggesting that the cells undergo differentiation immediately after neuronal differentiation has been induced. Although the number of cells increased upon the induction of differentiation, the cells subsequently showed a growth rate reduction and focused on the differentiation process.

 Capabilities of adult stem cells to differentiate into cells from different germ layers or cell lineages are known as transdifferentiation. Stem cells from bone marrow which originated from mesoderm were shown to be able to differentiate into liver, lung, gastrointestinal tract, and skin cells which derived from endoderm and mesoderm [51]. Peripheral blood stem cells (hematopoietic stem cells) also were found able to differentiate into mature cells which were not originated from hematopoietic cells such as liver, skin epithelial, and gastrointestinal tract cells [52]. Changes that occur to cell microenvironments such as addition of certain growth or differentiation factors during *in vitro* cell culture were found to be able to induce transdifferentiation [53]. Deletion of serum also contributed to this transdifferentiation since addition of serum in the medium allowed growth and maintenance of cells and prevented embryonic stem cells differentiation into neuronal cells [54]. In this study, the absence of serum and growth factors during culture may lead to DPSCs transdifferentiation into neuron-like cells.

## 5. Conclusions

Dental pulp stem cells are induced to differentiate into neuronal cells when cultured in serum- and growth factor-free medium.

## Figures and Tables

**Table 1:** Primers involved in RT-PCR experiments.

Gene/accession no.	Primers	Sequences	Expected product size (bp)	Annealing temperature (°C)
*Cd146* (NM_023061)	Forward	5′-GGACCTTGAGTTTGAGTGG-3′	479	60
	Reverse	5′-CAGTGGTTTGGCTGGAGT-3′		
*Cd166* (NM_009655)	Forward	5′-AACATGGCGGCTTCAACG-3′	630	61
	Reverse	5′-GACGACACCAGCAACGAG-3′		
*Nestin* (NM_016701)	Forward	5′-CGCTCGGGAGAGTCGCTT-3′	215	64
	Reverse	5′-CCAGTTGCTGCCCACCTTC-3′		
*Gapdh* (NM_008084)	Forward	5′-CAACGGCACAGTCAAGG-3′	717	62
	Reverse	5′-AAGGTGGAAGAGTGGGAG-3′		
*Tub3* (NM_023279)	Forward	5′-ACGCATCTCGGAGCAGTT-3′	125	61
	Reverse	5′-CGGACACCAGGTCATTCA-3′		
*Cd31* (NM_001032378.1)	Forward	5′-GGTCTTGTCGCAGTATCAG-3′	355	58
	Reverse	5′-ATGGCAATTATCCGCTCT-3′		
